# Hydrogen Peroxide Probes Directed to Different Cellular Compartments

**DOI:** 10.1371/journal.pone.0014564

**Published:** 2011-01-21

**Authors:** Mikalai Malinouski, You Zhou, Vsevolod V. Belousov, Dolph L. Hatfield, Vadim N. Gladyshev

**Affiliations:** 1 Department of Biochemistry and Redox Biology Center, University of Nebraska, Lincoln, Nebraska, United States of America; 2 Division of Genetics, Harvard Medical School, Brigham and Women's Hospital, Boston, Massachusetts, United States of America; 3 Center for Biotechnology, University of Nebraska, Lincoln, Nebraska, United States of America; 4 Institute of Bioorganic Chemistry, Russian Academy of Sciences, Moscow, Russia; 5 Molecular Biology of Selenium Section, Laboratory of Cancer Prevention, Center for Cancer Research, National Cancer Institute, National Institutes of Health, Bethesda, Maryland, United States of America; Instituto de Química - Universidade de São Paulo, Brazil

## Abstract

**Background:**

Controlled generation and removal of hydrogen peroxide play important roles in cellular redox homeostasis and signaling. We used a hydrogen peroxide biosensor HyPer, targeted to different compartments, to examine these processes in mammalian cells.

**Principal Findings:**

Reversible responses were observed to various redox perturbations and signaling events. HyPer expressed in HEK 293 cells was found to sense low micromolar levels of hydrogen peroxide. When targeted to various cellular compartments, HyPer occurred in the reduced state in the nucleus, cytosol, peroxisomes, mitochondrial intermembrane space and mitochondrial matrix, but low levels of the oxidized form of the biosensor were also observed in each of these compartments, consistent with a low peroxide tone in mammalian cells. In contrast, HyPer was mostly oxidized in the endoplasmic reticulum. Using this system, we characterized control of hydrogen peroxide in various cell systems, such as cells deficient in thioredoxin reductase, sulfhydryl oxidases or subjected to selenium deficiency. Generation of hydrogen peroxide could also be monitored in various compartments following signaling events.

**Conclusions:**

We found that HyPer can be used as a valuable tool to monitor hydrogen peroxide generated in different cellular compartments. The data also show that hydrogen peroxide generated in one compartment could translocate to other compartments. Our data provide information on compartmentalization, dynamics and homeostatic control of hydrogen peroxide in mammalian cells.

## Introduction

Reactive oxygen species (ROS) are often viewed as toxic compounds that damage cellular components and may lead to cell death. However, at physiological concentrations, they are essential intermediates involved in signaling and redox homeostasis [1], [2]. Several cellular processes are known to produce ROS, and specific enzymatic systems for generation and detoxification of hydrogen peroxide (H_2_O_2_) have been discovered. Mitochondria are an important source of ROS in mammalian cells. Superoxide (O_2_^−^) is mainly produced from complexes I and III and is rapidly dismutated to hydrogen peroxide and oxygen by superoxide dismutases [3]. Signaling events trigger ROS generation by NADPH oxidases (NOX) [4], [5]. A number of extracellular stimuli have been shown to generate H_2_O_2_ through NOXs [2] and many other processes and enzymes are also known to generate ROS (e.g., fatty acids oxidation). ROS, when produced in cells, can be removed by intracellular antioxidant systems and provoke damage to biomolecules. Measurement of relative contributions of various processes to ROS generation is challenging. For example, complex I in mitochondria has 2 sites for O_2_^−^ production, which are differentially affected at various physiological states [6]. In addition, the half-life of most ROS is short and concentrations are small, making it difficult to monitor their formation and removal [7], [8].

H_2_O_2_ is one of more abundant ROS in cells [2]. This compound has a dual role serving both as a toxic oxidant and as an essential signaling molecule that regulates cellular biological processes. Specific properties of H_2_O_2_ are mainly determined by its reactivity, redox potential and relative stability (as compared to other ROS) in cells and ability to pass through membranes [1], [8], [9]. These features of H_2_O_2_ allow it to serve as an important intra- and intercellular second messenger in signaling events and to control homeostatic redox state [10]. It was shown that endogenously produced H_2_O_2_ directly contributes to a signaling response. For example, H_2_O_2_ plays a role in signaling induced by platelet-derived growth factor (PDGF) [11], [12], epidermal growth factor (EGF) [13] and hormonal regulation.

To protect cells from oxidative damage, cells evolved several systems that directly or indirectly regulate hydrogen peroxide levels, mostly reducing or disproportionating this compound. H_2_O_2_ detoxification systems include both enzymatic (catalase, glutathione peroxidases, peroxiredoxins) and non-enzymatic (glutathione, vitamins A, C, and E, bilirubin) systems [6], [9]. Hydrogen peroxide is often generated in specific tightly regulated sites. Some NADPH oxidases that generate H_2_O_2_ in response to signaling events are targeted to specific cellular microdomains of plasma membranes by lipid rafts [14]. It is often thought that hydrogen peroxide can cross membranes [15], but recent studies have found that some membranes are poorly permeable to it [15], [16], [17]. It is proposed that specific aquaporins facilitate H_2_O_2_ transport through membranes [7]. Membrane lipid composition can also influence hydrogen peroxide diffusion across membranes. Yeast ergosterol biosynthesis mutants showed reduced membrane permeability to hydrogen peroxide [9]. Serving various functions, cellular organelles maintain redox control unique to each compartment and may also influence membrane permeability to H_2_O_2_ [18]. Plasma membrane was also shown to change permeability under certain conditions [15], [16], [17].

Cellular response to H_2_O_2_ depends on its subcellular location and concentration. Significant progress in detection of ROS in cells under different conditions has been achieved recently. Fluorescent chemical dyes (2′-7′-dichlorodihydrofluorescein diacetate (DCFDA), dihydrorhodamine 123 (DHR), dihydrocyanine) are routinely used for detection of ROS in cells. New generation of boronate switch multicolor H_2_O_2_ sensors has been synthesized. They could potentially be used for simultaneous detection of H_2_O_2_ in several compartments [19]. Some ROS sensitive probes could be delivered to mitochondria [20] or targeted to a specific intracellular site by protein tags [21]. Ratiometric sensors using fluorescent proteins [22], [23] and chemiluminiscent nanoparticles [24] have also been developed for detection of ROS in cells.

In this work, we utilized a recently developed H_2_O_2_ genetically encoded biosensor HyPer. Its design is based on OxyR, a natural bacterial H_2_O_2_ sensor/transcription factor [23]. Upon oxidation of the OxyR regulatory domain, a sulfenic acid is formed on C199 which is further resolved with C208 to form a disulfide bond [11], [25]. The disulfide can then be reduced with DTT *in vitro* or with a thiol-disulfide oxidoreductase *in vivo*. This property provides HyPer with the ability to reversibly change redox state depending on the local peroxide level in cells. Several reports show successful application of HyPer to track intracellular redox changes and redox signaling events [26], [27], [28], [29]. We targeted HyPer to various cellular compartments and examined its redox state in mammalian cells. We also compared sensitivity of HyPer with other available fluorescent ROS indicators.

## Results

### Characterization of HyPer response in HEK293 cells

**Figure 1:**
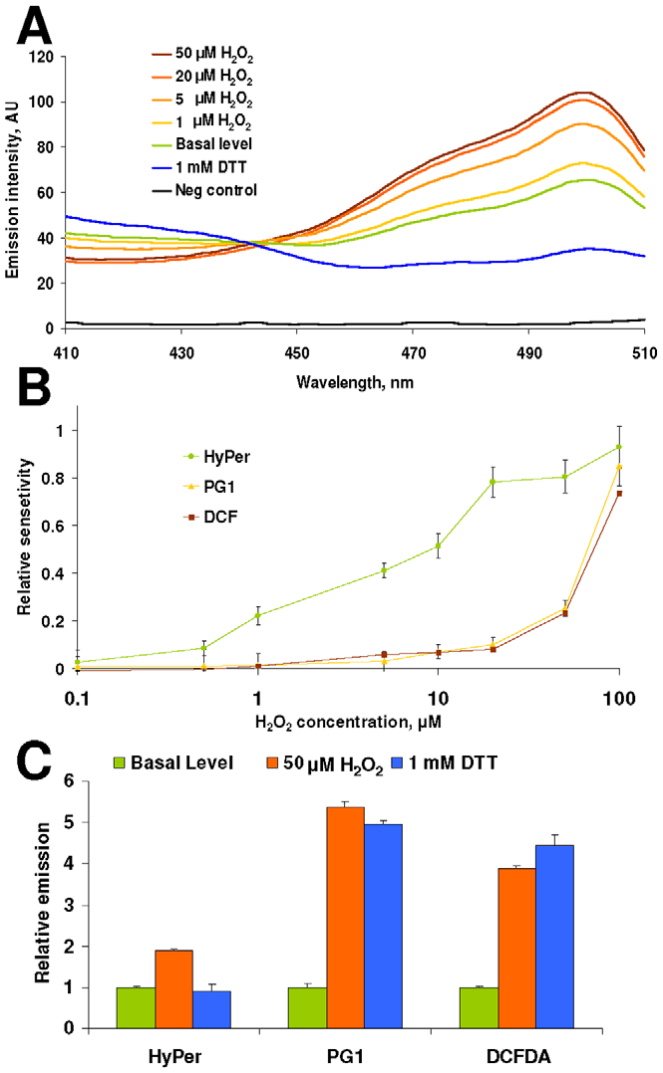
Response of HyPer expressed in HEK 293 cells to H_2_O_2_. (A) Fluorescence excitation spectra were recorded from HEK 293 cells expressing HyPer 3 min after indicated concentrations of H_2_O_2_ were added to cells or 30 min after DTT treatment. Untransfected cells were used as a negative control. Emission was recorded at 530 nm. (B) Sensitivity of various peroxide sensors to H_2_O_2_ treatment. Emission was recorded at 530 nm for HyPer and DCF and at 510 nm for PG1. (C) Probing H_2_O_2_ with various fluorescent sensors in HEK 293 cells oxidized with H_2_O_2_ (5 min) or reduced with DTT (30 min). All data represent mean of 3 measurements ± standard deviation.

HyPer was designed as a ratiometric bioindicator specific for H_2_O_2_ [23]. Following exposure of HEK 293 cells expressing HyPer in the cytosol to different concentrations of H_2_O_2_, fluorescence excitation spectra at the emission wavelength of 530 nm showed reciprocal changes in the areas near 420 and 500 nm (Figure 1A). As the relative ratio at these two wavelengths is less dependent on HyPer expression levels in cells, it provides a useful tool to examine transient changes in intracellular H_2_O_2_ levels. Treatment of HEK 293 cells with 1 mM DTT decreased fluorescence at 500 nm and increased fluorescence at 420 nm, indicating that HyPer was not fully reduced in HEK 293 cells. HyPer responded to micromolar concentrations of H_2_O_2_ added to the suspension of HEK 293 cells (Figure 1B). Since the intracellular concentration of H_2_O_2_ might be much lower (due to a gradient of this compound across the plasma membrane and scavenging activities of cellular redox systems), HyPer likely senses low micromolar concentrations of H_2_O_2_. At higher concentrations of H_2_O_2_, the HyPer response became saturated (Figure 1B). The sensitivity range of HyPer in HEK 293 cells was between 1 and 50 µM H_2_O_2_ added to cell culture medium (Figure 1B).

We further compared the H_2_O_2_ response of HyPer to those of previously described H_2_O_2_-sensitive dyes. DCF and PG1 showed more dramatic increases in fluorescence than did HyPer upon 50 µM H_2_O_2_ treatment (Figure 1C). However, HyPer detected lower levels of H_2_O_2_ (Figure 1B). In addition, DCF and PG1 responded irreversibly, whereas HyPer showed a reversible response following addition of 1 mM DTT to peroxide-treated cells.

### H_2_O_2_ in various cellular compartments

**Figure 2:**
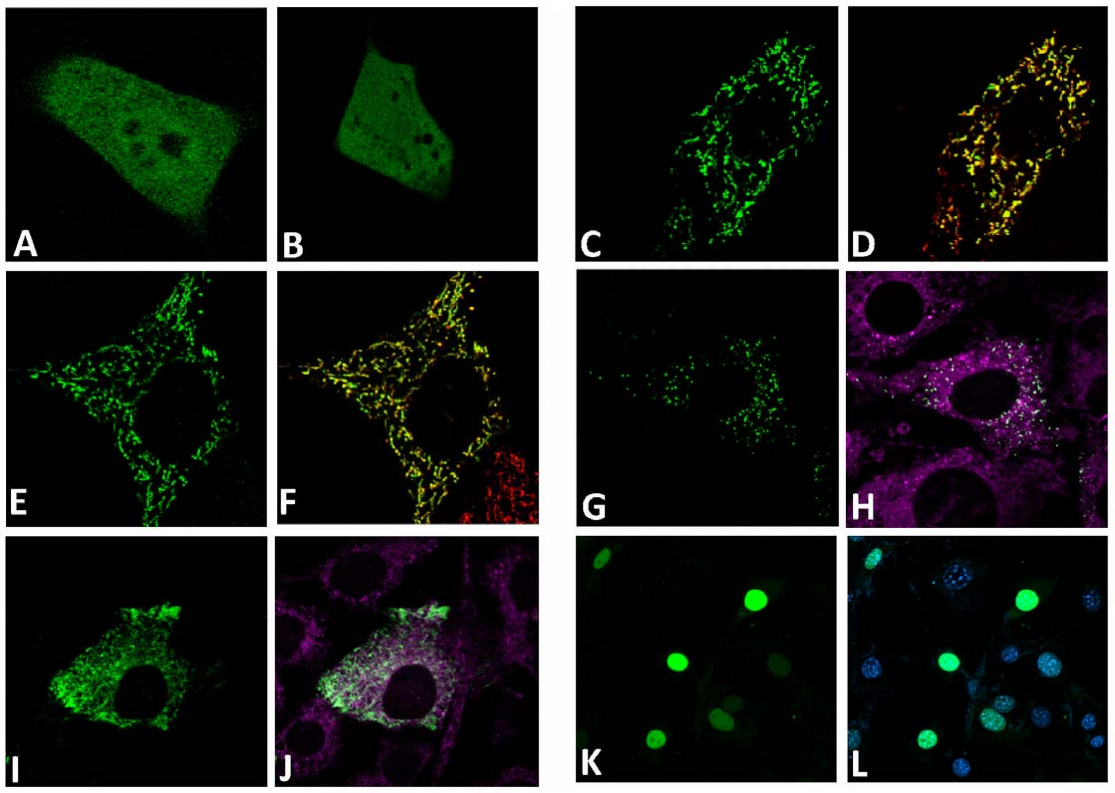
Intracellular localization of HyPer targeted to different cellular compartments in NIH 3T3 cells. Panels represent GFP (A) and HyPer expressed in the cytosol (B), mitochondria (C, D), mitochondrial IMS (E, F), peroxisomes (G, H), the ER (I, J), and the nucleus (K, L). Panels D, F are merged images with Mito-Tracker Red staining. Panels J, H show staining with ER-Tracker Blue, and L with Hoechst 34580. Arrows show mitochondrial IMS.

To examine the response to H_2_O_2_ treatment within various cellular compartments, HyPer was cloned, using signal peptides and/or retention sequences, to mitochondria, mitochondrial IMS, endoplasmic reticulum (ER), and peroxisomes. Figure 2 shows localization of the HyPer forms targeted to various compartments in comparison with corresponding organelle probes: Mito-Tracker Red (Figure 2D, F) and ER-Tracker Blue-White (Figure 2J, H). HyPer targeted to IMS co-localized with Mito-Tracker (Figure 2E and F). However, the fluorescence signals of the peroxide sensor and the marker were slightly different, with the IMS HyPer fluorescence surrounding the Mito-Tracker signal. This is consistent with the IMS localization of the sensor. Hyper was also targeted to the nucleus by cloning duplicated SV40 antigen nuclear localization signal (NLS) to the N-terminus of the sensor. Nuclear localization was verified by colocalizing HyPer with Hoechst 34580 nuclear probe (Figure 2L).

**Figure 3:**
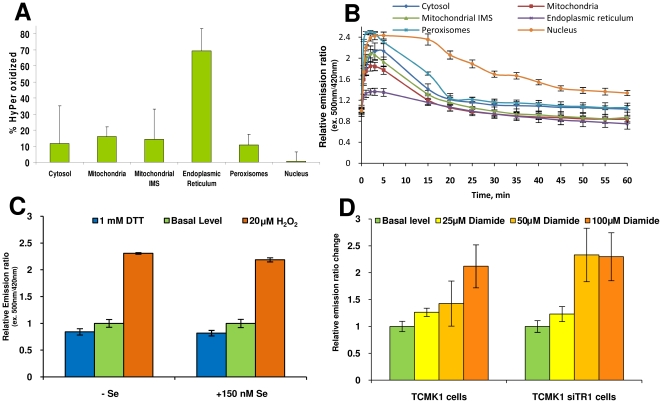
Redox state of HyPer targeted to different intracellular compartments. (A) Percentage of oxidized HyPer in cellular compartments. To estimate percentage of oxidized HyPer, we recorded fluorescence from HEK 293 cells stably expressing HyPer obtained under basal, reducing (30 min, 1 mM DTT), and oxidizing (3 min, 50 µM H_2_O_2_) conditions. (B) Dynamics of intracellular response of HEK 293 cells to 5 µM H_2_O_2_. (C) Redox state of HyPer under conditions that lead to the reduced expression of selenoproteins in HEK 293 cells. (D) TR1 knockdown TCMK cells treated with diamide. TR1 knockdown and control TCMK cells stably expressing HyPer in cytosol were treated for 30 min with indicated concentrations of diamide. Data represent mean of 3 measurements, ± standard deviation.

To examine kinetics of HyPer oxidation and reduction in mammalian cells, a time course experiment was carried out. 5 µM H_2_O_2_ was added to HEK 293 cells transfected with compartment-specific forms of HyPer, and fluorescence excitation spectra were recorded at 0.5, 1, 2, 3, 5 and up to 60 min following treatment (with 5 min increments) (Figure 3B). In each of the examined compartments, after a rapid increase in fluorescence intensity, the signal slowly decreased, often to the level even lower than the initial fluorescence. This effect is likely due to partial bleaching of cells and perhaps elevated activity of H_2_O_2_ scavenging systems in treated cells. Peroxisomes were the most responsive and the ER was the least responsive to H_2_O_2_ due to the fact that HyPer in the ER was mainly oxidized. HyPer targeted to the nucleus remained in the oxidized state longer than in other compartments. However, the overall changes in HyPer fluorescence were similar in each compartment. Using TableCurve analytical software, we described each data series with the following equation: *y = a+bx+cx^1.5^+dx^2^+ex^0.5^*, where x is time in minutes, y is emission intensity ratio, and parameters *a*, *b*, *c*, *d*, and *e* were calculated for each cellular compartment separately. Coefficient of determination for curves was between 0.95 and 0.99. We then calculated the time of maximal response and the time needed for cells to return to basal levels (Table 1). H_2_O_2_ reached the maximal levels in all compartments 2 and 4 min following addition of H_2_O_2_. The longest time to return to the basal level was observed for the nucleus, and the shortest for mitochondria and the ER (Figure 3B).

**Table 1:** Parameters of HyPer response to H_2_O_2_ in cellular compartments of HEK 293 cells.*

Cellular compartment	Time of max response, min	Max response, ratio change	Time to return to basal level, min
Cytosol	3.03±0.11	2.06±0.14	50.1±20.4
Mitochondria	2.42±0.13	1.87±0.12	24.2±3.63
Mitochondrial IMS	2.42±0.09	2.06±0.08	30.6±8.18
Endoplasmic Reticulum	3.03±0.10	1.37±0.06	26.1±6.06
Peroxisomes	2.42±0.11	2.55±0.03	59.9±1.81
Nucleus	4.24±0.61	2.53±0.03	96.5±4.50

* based on the data shown in Figure 3B.

HEK 293 cells stably expressing various forms of HyPer were then prepared and treated with 1 mM DTT or 50 µM H_2_O_2_ to generate HyPer spectra in fully reduced and fully oxidized conditions, respectively. In cytosol, mitochondria, mitochondrial IMS and peroxisomes, the HyPer fluorescence ratio changed significantly when cells were treated with H_2_O_2_, and only slightly when cells were exposed to DTT. Thus, HyPer was mostly in the reduced state in these compartments (Figure 3A). However, in the ER, HyPer reacted mostly to DTT, whereas the response to H_2_O_2_ was small, suggesting that the sensor occurred in the ER in the oxidized form. By quantifying fluorescence ratio changes under oxidizing and reducing conditions, we calculated the amounts of oxidized and reduced forms of HyPer in untreated conditions (Table S2). In cytosol, mitochondria, mitochondrial IMS and peroxisomes, 10-16% HyPer was in the oxidized form, whereas in the ER, ∼70% HyPer was oxidized. The most reducing environment was observed in the nucleus (Figure 3A, Table S2). The data are consistent with the idea that the majority of cellular compartments (except the ER) have a low, but pronounced H_2_O_2_ tone. In contrast, the environment in the ER supports oxidation of HyPer, either due to higher peroxide tone or efficient disulfide bond formation.

### HyPer response in selenium-deficient cells

Mammals contain 24–25 selenoproteins, many of which are oxidoreductases [30]. The most abundant mammalian selenoprotein is glutathione peroxidase 1 (GPx1), which is a major enzyme that reduces H_2_O_2_ to water in the cytosol and uses GSH as electron donor. To test the role of GPx1 in H_2_O_2_ homeostasis, we decreased its levels (as well as the levels of other selenoproteins) in HEK 293 cells by growing these cells in a selenium-deficient medium (Figure S1). Figure 3C shows the HyPer response in cytosol before and after addition of 20 µM H_2_O_2_ and subsequent reduction with 1 mM DTT. No significant difference in fluorescence was observed between selenium-deficient and -supplemented cells. Using HyPer targeted to different compartments we also tested the redox state of this sensor in other compartments in cells subjected to selenium deficiency (Figure S2). Our data suggest that the decrease in GPx1 levels in HEK 293 cells by selenium deficiency did not alter H_2_O_2_ levels in the cellular compartments.

Additionally, we investigated the cytosolic peroxide-based redox regulation in mouse embryonic stem cells derived from GPx1 knockout mice. These cells were transfected with the cytosolic HyPer construct and treated with H_2_O_2_ or DTT. The redox state of cytosolic HyPer was not affected in GPx1 knockout cells (Figure S3). We further extended the study to analyze the consequences of knockdown of two other selenium-related gene products in mammalian cells: SECIS-binding protein 2 (SBP2) and thioredoxin reductase 1 (TR1). Knockdown of SBP2 in mouse Hepa1-6 cells only slightly oxidized cytosolic HyPer (Figure S3).

TR1 is a cytosolic NADPH-dependent FAD-containing selenoprotein that maintains the reduced state of thioredoxin, which in turn keeps cysteine residues in cellular proteins in the reduced state. These two proteins constitute one of two major redox regulatory systems in cells (together with the glutathione system) [31]. We compared HyPer response of TR1 knockdown [32] and control TCMK1 cells (Figure S4) to diamide treatments. The redox state of the sensor did not change significantly upon down-regulation of TR1. However, TR1 knockdown cells showed a HyPer response at lower diamide concentrations, whereas control cells did not respond to these diamide levels (Figure 3D). As diamide can non-specifically oxidize free thiol groups in proteins, this assay illustrates how HyPer can be used to examine the compromised function of the thioredoxin system. Using this model we tested the effect of TR1 knockdown in various cellular compartments (Figure S5). HyPer targeted to mitochondria, mitochondrial IMS and the nucleus showed a higher response to oxidation by diamide in siTR1 TCMK1 cells. It should be noted that TR1 exists in multiple forms, generated by alternative first exon splicing, that may result in TR1 occurring in different cellular compartments.

### Generation of H_2_O_2_ in various cellular compartments

**Figure 4:**
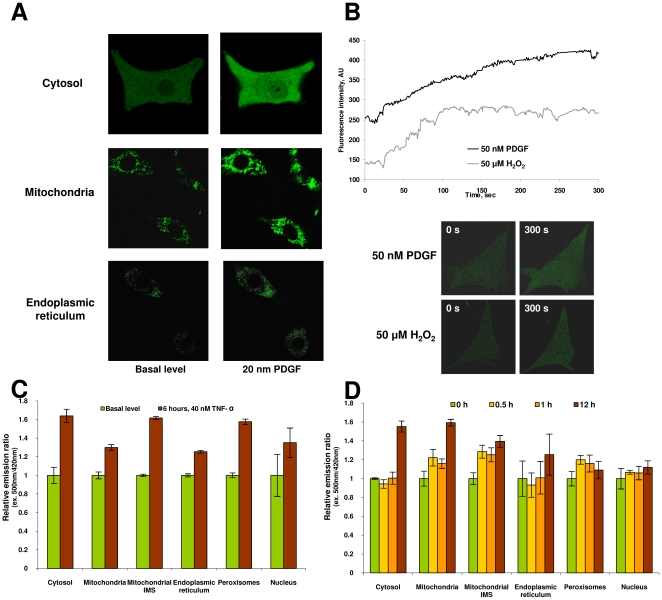
Changes in HyPer redox state following redox perturbations. (A) Response of HyPer expressed in various compartments of NIH 3T3 cells to PGDF. NIH 3T3 cells transiently expressing HyPer were stimulated with 20 nM PDGF for 10 min. Images obtained before and after treatment are shown. (B) Dynamics of intracellular fluorescent HyPer response in NIH 3T3 cells treated with 20 nM PDGF or 50 µM H_2_O_2_. Graphs represent mean fluorescence intensity of one representative cell in 5 min time-lapse. (C) Generation of H_2_O_2_ during TNF-α signaling in various compartments of HEK 293 cells. HEK 293 cells were incubated with 40 ng/ml TNF-α for 6 h. Fluorescence was recorded at emission 530. (D) Inhibition of Complex I of mitochondrial ETC leads to H_2_O_2_ generation in HEK 293 cells. Data represent mean of 3 measurements, ± standard deviation.

Treatment of cells with PDGF is known to result in ROS production [12], [13]. We monitored changes in the redox state of HyPer in various cellular compartments upon PDGF stimulation of NIH 3T3 cells (Figure 4A,B). Confocal microscopy images showed an increased fluorescence of HyPer in cytosol and mitochondria following addition of PDGF (Figure 4A). Interestingly, time course analysis showed differences in cytosolic HyPer fluorescence changes between 50 µM H_2_O_2_ and 50 nM PDGF treatments (Figure 4B). The H_2_O_2_ response was fast and saturated between 1 and 2 min, whereas the PDGF response was slower and continuously on the rise. These data suggest that H_2_O_2_ added extracellularly quickly penetrates the cell membrane and directly oxidizes HyPer, whereas PDGF acts to generate H_2_O_2_ intracellularly following a signaling cascade, resulting in a delayed response.

Tumor necrosis factor alpha (TNF-α) is a proinflammatory cytokine that is also involved in apoptosis, proliferation, differentiation, and tumorigenesis [33]. We examined H_2_O_2_ metabolism in cellular compartments following TNF-α treatment (40 ng/ml, 6 h) of HEK 293 cells (Figure 4C). All compartments tested showed HyPer response. The most significant changes were registered in cytosol. Mitochondrial matrix showed a lower response. This observation is consistent with the idea that redox signaling pathways may be utilized to generate H_2_O_2_ at various intracellular sites.

To further examine the sites of H_2_O_2_ generation in mitochondria, an electron transport chain (ETC) inhibitor, rotenone, was used. Rotenone inhibits ubiquinone reduction by binding to Complex I, inducing superoxide production [34] and raising NADH levels [35]. ROS generated by rotenone in cells are converted to H_2_O_2_ and can be detected by HyPer in IMS and other compartments (Figure 4D). Mitochondrial matrix and IMS HyPer responded rapidly to rotenone treatment (0.5 h). Upon prolonged rotenone treatment, the major increase in H_2_O_2_ was detected in the matrix. After 12 h inhibition of ETC, HyPer was more oxidized in mitochondria and IMS than in cytosol. Cytosolic HyPer oxidation was observed only after 12 h treatment with rotenone. This observation may also serve as an additional indirect evidence of changes in mitochondrial membrane permeability to ROS induced by rotenone [36], [37]. Upon ETC inhibition, most changes occur on the mitochondrial membrane. Other compartments tested (peroxisomes, the ER and the nucleus) (Figure 4D) did not change the redox state of the sensor.

The ER is a major site of folding of secreted proteins. Oxidizing environment in this compartment is favorable to disulfide bond formation in proteins [38]. Two known systems responsible for disulfide bond formation in the ER are Ero1-PDI and sulfhydryl oxidase QSOX1. PDI and Ero1 represent the major pathway for disulfide bond formation in the ER [38]. Ero1 accepts reducing equivalents from PDI and other substrates and transfers them to molecular oxygen, thus producing H_2_O_2_ [38]. We tested if siRNA knockdowns of Ero1, QSOX1 or both affect HyPer redox state in the ER of HEK 293 cells (Figure S6). No significant changes were observed for either ER or cytosolic forms of HyPer (Figure S7). Additionally, HEK293 cells were treated with tunicamycin, thapsigargin or brefeldin A for 6 h. HyPer redox state did not change in response to these ER stressors (Figure S8).

## Discussion

In this work, we examined the processes that lead to generation and removal of H_2_O_2_ in various cellular compartments using a genetically encoded bioindicator, HyPer. This sensor appears to be more sensitive than the previously described redox sensor, roGFP [22]. It was almost fully oxidized by 50 µM H_2_O_2_ in cell culture media and sensed low micromolar H_2_O_2_ in the cell. HyPer is characterized by EC_50_ = 7.97±0.48 µM for H_2_O_2_, which is almost 25 times lower than that for roGFP. H_2_O_2_ sensitive dyes, PG1 [39] and DCF, showed more significant changes in fluorescence intensity than HyPer. Unlike other probes HyPer showed a reversible response and could potentially be used to examine both oxidative and reductive phases of redox treatments. More importantly, HyPer sensed lower levels of H_2_O_2_ than these chemicals. On the other hand, one consideration in using HyPer is its signal dependence on the pH of the environment [23]. However, it has been previously noted that HyPer response in the pH range is similar to other cpYFP-based sensors (e.g., Pericam) [40].

Thus, HyPer fills an important niche in examining homeostatic control of H_2_O_2_ due to a combination of sensitivity, specificity and reversibility. These properties are very useful in examining the role of H_2_O_2_ in redox signaling and homeostasis. HyPer has been proved to be a useful probe to monitor levels of H_2_O_2_ during signaling events [27], [41] and metabolic processes [39]. Recently, it was expressed in zebrafish to detect H_2_O_2_ in wounds [42].

Targeting HyPer to various cellular compartments provided important insights into H_2_O_2_ metabolism and redox environment of these compartments. Cytosol, mitochondria, nucleus and peroxisomes showed mostly reduced HyPer, yet some oxidized HyPer was detected, suggesting the occurrence of low levels of H_2_O_2_. Despite the reducing environment in the cytosol, some proteins are regulated by thiol-based redox switches [43] and regulatory disulfide bonds are formed in cytosolic proteins.

Another ROS genetically encoded sensor, roGFP, showed the lowest redox state in mitochondria [44]. This sensor mainly reflects reduced glutathione levels, which may shadow elevation in H_2_O_2_ levels. In contrast to roGFP [22], HyPer did not significantly change the redox state upon GSH depletion with buthionine sulfoximine (BSO). In the current work, we observed the most reduced HyPer in the nucleus. This is consistent with the signaling role of H_2_O_2_ for many transcriptional factors [45], [46] and the need for the cell to protect its DNA from oxidative damage [47], [48]. Recently, an increased level of GSH was observed in the nucleus in NIH 3T3 cells [49]. It is also proposed that GSH is required to modulate thiol/redox status of proteins in the nucleus and control chromatin compacting [50]. Very low peroxide tone maintained in the nucleus is likely required for cell physiology. It is known that H_2_O_2_ is needed for cell signaling and its low or high levels may disrupt signaling pathways [1], [12]. The observation of the peroxide tone is consistent with this idea.

In contrast to most cellular compartments, HyPer in the ER was largely in the oxidized state. At present, the molecular basis for HyPer oxidation is not clear. In a recent study, redox control in yeast ER was examined using roGFP. This redox-sensitive GFP was 96.9% oxidized [51]. Two enzymatic systems are known that are involved in disulfide bond formation in the ER: Ero1-PDI and QSOX1. Sulfhydryl oxidases Ero1 and QSOX1 are FAD-binding enzymes that transfer reducing equivalents to molecular oxygen generating H_2_O_2_. The ER could be the major site of peroxide generation in cells, and the redox state of HyPer in this compartment could be the reflection of high peroxide levels. Alternatively, HyPer could be directly oxidized in the ER by PDI or other disulfide formation proteins. In this case, the redox state of HyPer could reflect robust disulfide bond formation. Currently, these two possibilities are difficult to distinguish experimentally. For example, downregulation of disulfide bond formation machinery may be expected to lead to both decreased peroxide formation and decreased oxidative folding in the ER. However, we observed no significant difference in the oxidation state of HyPer in case of single or simultaneous knockdowns of Ero1 and QSOX1 in HEK 293 cells. Independent of the mechanism of HyPer oxidation, HyPer targeted to this compartment provides a novel probe for examining redox homeostasis in the ER.

Recently, it was discovered that disulfide bonds are actively formed in the mitochondrial IMS [52]. This process is similar to that in the ER and found to be the mechanism for targeted retention of proteins in the IMS. It is thought that the IMS has a somewhat oxidizing environment [53]. However, our results indicate a reducing environment in the IMS with regard to peroxide. Such environment is thought to be maintained mainly through GSH [54]. However, GSH levels estimated by roGFP targeted to the IMS were not significantly different from those in the cytosol [55]. This observation suggests that disulfide bonds are formed in the IMS under reducing conditions. Previous studies showed that ROS produced by Complex I are mainly directed to the mitochondrial matrix [56] and by complex III to both sides of the inner membrane [57]. Redox-active import receptor Mia40 is responsible for disulfide bond formation in the IMS [58]. Mia 40 together with sulfhydryl oxidase Erv1 serve a function in the IMS similar to that of PDI-Ero1 in the ER and DsbA-DsbB in the bacterial periplasm. A redox relay system consisting of Erv1 and Mia 40 is activated by ETC inhibition thus stimulating disulfide bond formation in the IMS [58]. Prolonged inhibition of Complex I ETC changed the HyPer redox state most dramatically in the mitochondrial matrix. Inhibition of Complex I produced superoxide inside the mitochondrial matrix where it was converted to H_2_O_2_ by SOD [3]. Using HyPer, we established an important difference between the ER and the IMS. Whereas both compartments form disulfide bonds, the ER exhibits an oxidizing environment, and the IMS maintains a reducing environment.

Growth factor-based receptor signaling is at the heart of studies on redox signaling. There have been numerous reports showing that PDGF binding to its receptor leads to generation of H_2_O_2_ as part of the signaling process [2]. In our experiments, intercompartment differences in the distribution of H_2_O_2_ upon PDGF treatment were not distinguished, even though sensitivity of the sensor was sufficient to monitor peroxide changes. This suggests that H_2_O_2_ generated in one compartment is rapidly distributed to other cellular compartments. TNF-α is a commonly used cytokine for redox signaling studies [59]. TNF-α signaling leads to accumulation of unfolded proteins in the ER and to ER-associated protein degradation [60], and the resulting ROS can oxidize HyPer, suggesting a role of H_2_O_2_ in these processes. Upon treatment with TNF-α, cells are thought to oxidize mitochondrial, but not cytosolic thioredoxin [61].

Intracellular H_2_O_2_ detoxification systems can control the level of this oxidant. For example, H_2_O_2_ can be decreased by GPxs, catalase, peroxiredoxins and other proteins, and redox compounds [6], [9]. Cells grown on selenium deficient media and MEFs from GPx1 knockout mice showed unaltered levels of H_2_O_2_ in the cytosol under physiological conditions. GPx1 mice are known to grow, develop and reproduce normally under physiological conditions, but have increased sensitivity to paraquat treatment [62]. SBP2 is one of key components of the selenocysteine insertion machinery. SBP2 knockdown in cells leads to decreased levels of selenoproteins and its expression is induced upon oxidative stress [63]. Thioredoxin and glutathione systems are considered potentially as electron donors to SBP2, thus regulating redox homeostasis through selenoprotein biosynthesis. However, we did not observe significant changes in HyPer redox state in SBP2 knockdown cells. Perhaps, the SBP2 function can only affect peroxide homeostasis under severe oxidative stress or only cells with lower levels of other peroxide-detoxification systems.

Finally, cytosolic thioredoxin reductase [64] serves a key role in maintaining the reduced state of thioredoxin, which in turn controls the reduced state of cysteines in numerous proteins [31]. We studied how the decrease in TR1 expression affects the HyPer redox state in different cellular compartments. Differences in the HyPer response between TR1 knockdown and control cells were observed in the cytosol, mitochondria and the nucleus. As an important antioxidant enzyme, thioredoxin reductase 1, maintains a reducing environment in different compartments, including the nucleus [65]. Recently, it has been shown that mitochondrial thioredoxin reductase (TR3) can be localized to the cytosol through alternative splicing [66]. More importantly, cytosolic Trx1 can serve as a target for TR3.

In conclusion, by using the genetically encoded sensor, HyPer, we were able to examine generation and removal of H_2_O_2_ in various compartments of mammalian cells. Low levels of this compound occur in all cellular compartments. The nucleus maintains the lowest physiological level of H_2_O_2_ in the cell. H_2_O_2_ can translocate across mitochondrial membranes. One exception is the ER, where HyPer was mostly oxidized. This finding provides a novel tool to examine redox processes in this compartment. HyPer manifested great sensitivity and could register low micromolar concentrations of H_2_O_2_ added to cell culture.

## Materials and Methods

### Cloning of HyPer-based H_2_O_2_ biosensors targeted to various cellular compartments

Cytosolic and mitochondrial HyPer constructs were prepared as described [23]. For targeting HyPer to other cellular compartments, signal peptides and other targeting sequences were cloned to N- or/and C-termini of HyPer. The mitochondrial signal had two copies of the targeting signal of subunit VIII of human cytochrome C oxidase [23]. The ER targeting and retention sequences were from mouse SelM (accession number AY043488). Forward (Pr1) and reverse (Pr2) primer sequences used to generate the vector are listed in Table S1. All cloning and sequencing primers were supplied by Integrated DNA Technologies (IDT, Coralville, IA, USA). The peroxisomal signal (PTS1) on HyPer was the tripeptide SKL placed at the C-terminus [67]. Primers Pr3 and Pr4 were used. To target HyPer to the mitochondrial intermembrane space (IMS), it was fused at the N-terminus with a partial sequence of mouse glycerol phosphate dehydrogenase 2. PCR products were digested at *Nhe1*/*BamH1* sites and ligated into the pEGFP-C3 vector (Clontech, Mountain View, CA, USA). To clone mouse glycerol phosphate dehydrogenase 2, mRNA was extracted from NIH 3T3 cells using RNeasy Mini Kit (Qiagen) and followed with RT-PCR (SuperScript III One-Step RT-PCR System, Invitrogen, Carlsbad, CA, USA) using Pr5 and Pr6 primers. The PCR product was digested at *Nhe1*/*BamH1* sites and ligated into HyPer-mito vector replacing the mitochondrial targeting signal. A duplicated nuclear localization signal (NLS) from SV40 large T antigen was used to target HyPer to the nucleus. It was cloned downstream of HyPer in two consecutive rounds of PCR using the same forward primer (Pr3) and two different reverse primers (Pr7 and Pr8). The product was digested and ligated back to the vector at *Nhe1/BamH1* sites.

### Cell lines and cell culture

Cells were cultured in Dulbecco Modified Eagle's Minimal Essential Medium (DMEM) (Invitrogen, Carlsbad, CA, USA) supplemented with 10% newborn calf serum (Invitrogen Carlsbad, CA, USA) and 5 mg/ml penicillin-streptomycin. HEK 293 cells (Invitrogen Carlsbad, CA, USA) were transfected with HyPer constructs using calcium-phosphate method. All other cell lines were transfected according to the manufacturer's instructions using FuGene HD (Roche Applied Science, Foster City, CA, USA) transfection reagent. Transfection mixtures were prepared at a 5∶2 ratio of FuGene HD to DNA. To compare the sensitivity of HyPer and fluorescent dyes, HEK 293 cells were stained with 6-carboxy-2′,7′-dichlorodihydrofluorescein diacetate (acetoxymethyl ester) (DCFDA) (Molecular Probes, Carlsbad, CA, USA) or Peroxy Green 1 (PG1) [39]. Decreased expression of GPx1 and other selenoproteins in HEK 293 cells was achieved by growing cells in selenium-deficient media. HEK 293 cells stably expressing HyPer were incubated for 48 h in DMEM containing 4 µg/ml insulin, 5 µg/ml transferrin, and 5 mg/ml penicillin-streptomycin. Selenium-supplemented medium was the same insulin/transferrin medium supplemented with 150 nM Na_2_SeO_3_.

### Cell imaging

Cultured NIH 3T3 cells on glass bottom dishes (MatTek Corporation, Ashland, MA, USA) were analyzed in a live cell chamber with 5% CO_2_ at 37°C 24–48 h following transfection using an inverted microscope with Olympus FV-500 confocal laser scanning system. Cells were starved for 12 h in serum-free medium prior to stimulation and image collection. To visualize the intracellular location of HyPer, cells were stained with Mito-Tracker Red, ER-Tracker Blue-White or Hoechst 34580 (Molecular Probes, Carlsbad, CA, USA) at a final concentration of 200 nM in DMEM for 20 min. Cells were washed twice with serum-free DMEM. For high resolution localization images cells were grown on coverslips and then fixed in 4% paraformaldehyde in PBS for 60 minutes then mounted on slides using mounting media, dried and imaged. HyPer fluorescence was collected with excitation using 488 nm laser line and images of markers was collected using 405 nm laser excitation for ER-Tracker and Hoechst 34580 and 543 nm laser line for Mito-Tracker Red.

### RNA interference in HEK 293 cells

Ero1 and QSOX knockdown experiments were performed using On-Target Plus SMARTpool siRNA reagents (Dharmacon, Lafayette, CO, USA). HEK 293 cells stably expressing HyPer were seeded on six-well plates. The following day, cells were transfected with 6 nM of either experimental siRNA or scrambled siRNA control, using DharmaFECT 1 reagent according to the manufacturer's instructions. Cells were incubated for 48 h, harvested and analyzed for HyPer redox state by fluorescence spectroscopy and for gene expression by RT-PCR. Cells deficient in thioredoxin reductase 1 were described previously [32].

### Fluorescence spectroscopy

HEK 293 cells stably expressing HyPer or stained with ROS sensitive dyes were trypsinized, washed twice in phosphate buffer saline (PBS), pH 7.4, and placed in a 1 ml cuvette or 96-well plate. Excitation spectra of HyPer expressed in cells were recorded using a Cary Eclipse fluorescence spectrophotometer (Varian, Palo Alto, CA, USA) with excitation at 410–510 nm, 5 nm slit, and emission at 530 nm, 5 nm slit. Cells stained with DCF were excited at 490 nm with emission at 530 nm and the PG1-stained cells were excited at 460 nm and their emission assayed at 510 nm, 5 nm slit. To examine redox properties of HyPer in cellular compartments, spectra were recorded from HEK 293 cells stably expressing various forms of HyPer, which were treated with either 1 mM DTT for 30 min or 50 µM of H_2_O_2_ for 5 min. Kinetics were studied using Gemini XPS fluorescence microplate reader (Molecular devices, Sunnyvale, CA, USA). HEK 293 cells were trypsinized, washed and diluted in PBS. Cell numbers were adjusted to 4×10^6^/ml using Countess automated cells counter (Invitrogen, Carsbad, CA, USA). Fluorescence intensity was recorded at 535 nm emission (with 530 nm cutoff filter) using 420 nm and 500 nm excitation filters. 96-well microplates were kept at 37°C and fluorescence intensity was recorded at various time points during 60 minutes after addition of 5 µM H_2_O_2._ To examine differences in response to H_2_O_2_, data series were analytically analyzed using curve fitting software, TableCurve 2D (Jandel Scientific, Bedfordshire, UK).

### Fluorescence-activated cell sorting

Cells transiently expressing various HyPer forms were trypsinized 24–48 h after transfection, washed twice in PBS (pH 7.4) and diluted in PBS to approximately 10^6^ cells/ml. Samples were analyzed by FACS Calibur (BD Biosciences, Franklin Lakes, NJ, USA) using 488 nm Argon laser (120 mW). Cells were incubated with either 1 mM DTT for 30 min or 50 µM of H_2_O_2_ for 5 min.

## Supporting Information

Figure S1 Downregulation of GPx1 expression in HEK 293 cells by selenium deficiency. HEK 293 cells were maintained for 48 h on a regular medium (10% NCS) (lane 1); selenium deficient medium (lane 2); and the same medium supplemented with 150 nM Na_2_SeO_3_ (lane 3). The upper panel shows an immunoblot assay with anti-GPx1 antibodies, and the lower panel protein staining with Coomassie Blue (for protein loading).
(0.29 MB TIF)

Figure S2 Redox state of HyPer targeted to different compartments under conditions that lead to the reduced expression of selenoproteins in HEK 293 cells. Cells expressing HyPer targeted to mitochondria, mitochondrial IMS, the endoplasmic reticulum, peroxisomes and nucleus were incubated for 48 h in DMEM containing 4 µg/ml insulin, 5 µg/ml transferrin, and 5 mg/ml penicillin-streptomycin. Selenium-supplemented medium was the same insulin/transferrin medium supplemented with 150 nM Na_2_SeO_3_. Cells were trypsinized, washed and diluted in PBS (pH 7.4). Cells were treated with 1 mM DTT for 30 min or 20 µM H_2_O_2_ for 3 min. Fluorescence intensity ratio was obtained using emission at 530 nm and excitations at 500 nm and 420 nm. Bars represent the average of 3 measurements, ± standard deviation.
(0.71 MB TIF)

Figure S3 Redox state of cytosolic HyPer expressed in different cell types. Percentage of oxidized HyPer in MEF cells derived from GPx1 knockout mice (left) and siRNA SBP2 Hepa1-6 stable cell line (right). Cells were trypsinized, washed and diluted in PBS (pH 7.4). Fluorescence intensity ratio was measured for untreated cells (basal conditions), treated with 1 mM DTT for 30 min (fully reduced state) or 100 µM H_2_O_2_ (oxidized state) for 5 min. Fluorescence intensity ratio was obtained using emission at 530 nm and excitations at 500 nm and 420 nm.
(0.23 MB TIF)

Figure S4 Expression of TR1 in TCMK1 cells. The upper panel shows immunoblot analysis of TR1 expression in TCMK1 cells (lane 1) and TCMK1 siTR1 cells (lane 2). The lower panel shows protein staining as a loading control.
(0.22 MB TIF)

Figure S5 Redox state of HyPer targeted to different compartments in TR1 knockdown TCMK1 cells treated with diamide. TR1 knockdown and control TCMK cells stably expressing HyPer in the cytosol were treated for 30 min with indicated concentrations of diamide. Fluorescence intensity ratio was obtained using emission at 530 nm and excitations at 500 nm and 420 nm. Data represent mean of 3 measurements, ± standard deviation.
(0.68 MB TIF)

Figure S6 Ero1 mRNA levels in knockdown cells. mRNA was isolated from Ero1 siRNA-transfected and scrambled siRNA-transfected HEK 293 cells and subjected to semi-quantitative RT-PCR. Lanes represent RNA isolated from cells transfected with scrambled siRNA (lanes 2 and 4) and Ero1 siRNA (lanes 3 and 5). Molecular weight markers are shown in lane 1.
(0.10 MB TIF)

Figure S7 Knockdown of Ero1 or QSOX1 does not affect redox state of HyPer in HEK 293 cells. HEK 293 cells stably expressing HyPer in the ER were transfected with control (non-targeting), Ero1 or QSOX1 siRNAs. Fluorescence intensity was recorded 48 h after transfection. In addition, cells were treated with 1 mM DTT and 100 µM H_2_O_2_ to determine the range of oxidation states of HyPer in cells. Data represent mean of 3 independent measurements.
(0.12 MB TIF)

Figure S8 HyPer response to activators of ER stress. HEK293 cells stably expressing HyPer in the ER were treated with 2 µg/µl tunicamycin, 5 µg/µl brefeldin A or 2 µM thapsigargin for 6 h. In addition, cells were treated with 1 mM DTT and 100 µM H_2_O_2_ to determine the range of oxidation states of HyPer in cells. Data represent mean of 3 measurements, ± standard deviation.
(0.12 MB TIF)

Table S1 Primers used to create constructs expressing HyPer targeted to various cellular compartments.
(0.01 MB DOCX)

Table S2 Redox state of HyPer in various cellular compartments of HEK 293 cells.
(0.01 MB DOCX)
