# Supplementary figures and images for: Hydrogen Peroxide Probes Directed to Different Cellular Compartments
Source: PLoS One. 2011 Jan 21;6(1):e14564. doi: 10.1371/journal.pone.0014564 (PMC3024970; doi:10.1371/journal.pone.0014564)

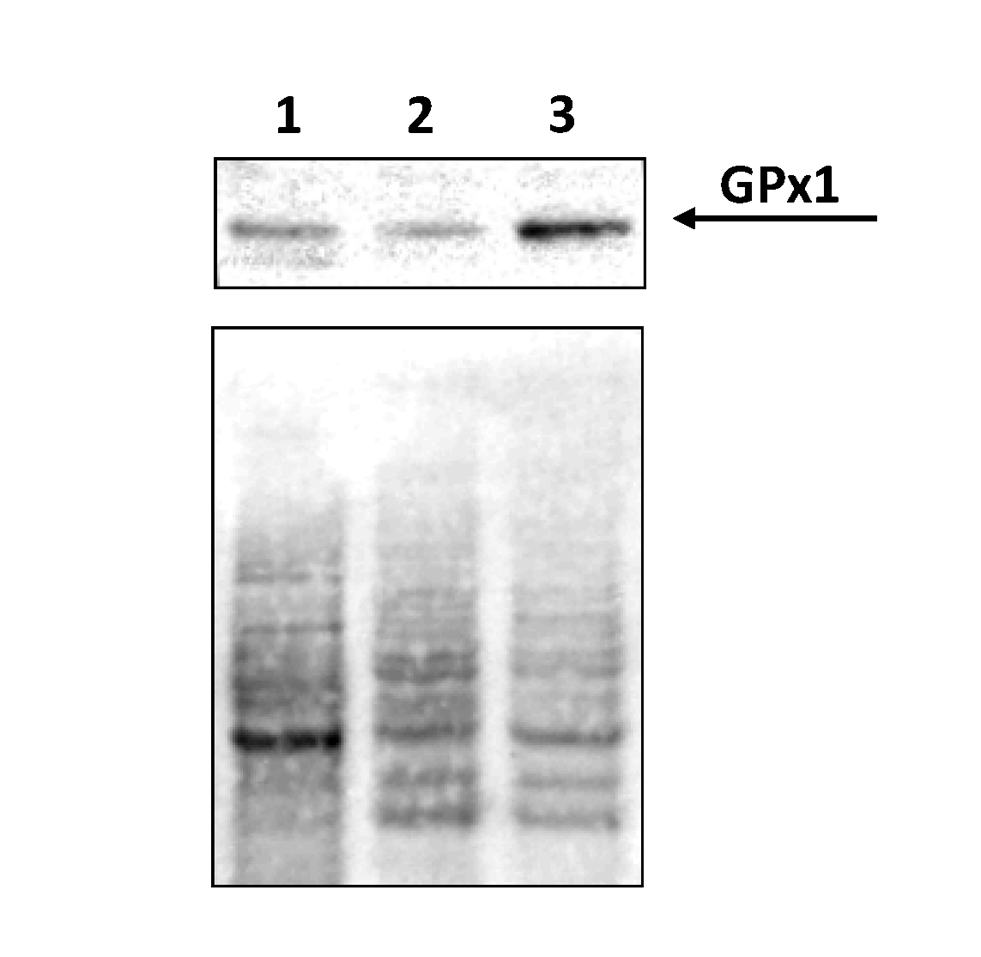

Supplement: Figure S1 — Downregulation of GPx1 expression in HEK 293 cells by selenium deficiency. HEK 293 cells were maintained for 48 h on a regular medium (10% NCS) (lane 1); selenium deficient medium (lane 2); and the same medium supplemented with 150 nM Na2SeO3 (lane 3). The upper panel shows an immunoblot assay with anti-GPx1 antibodies, and the lower panel protein staining with Coomassie Blue (for protein loading). (0.29 MB TIF) [file pone.0014564.s001.tif]

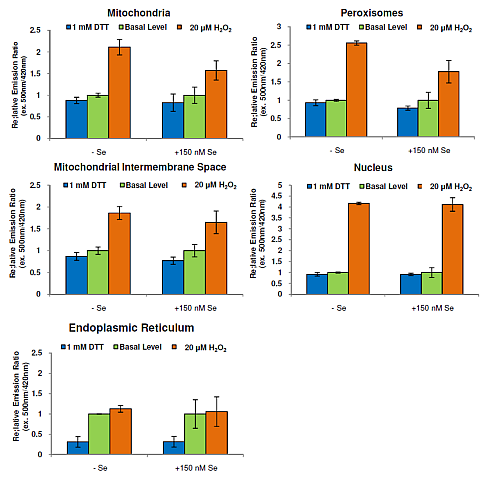

Supplement: Figure S2 — Redox state of HyPer targeted to different compartments under conditions that lead to the reduced expression of selenoproteins in HEK 293 cells. Cells expressing HyPer targeted to mitochondria, mitochondrial IMS, the endoplasmic reticulum, peroxisomes and nucleus were incubated for 48 h in DMEM containing 4 µg/ml insulin, 5 µg/ml transferrin, and 5 mg/ml penicillin-streptomycin. Selenium-supplemented medium was the same insulin/transferrin medium supplemented with 150 nM Na2SeO3. Cells were trypsinized, washed and diluted in PBS (pH 7.4). Cells were treated with 1 mM DTT for 30 min or 20 µM H2O2 for 3 min. Fluorescence intensity ratio was obtained using emission at 530 nm and excitations at 500 nm and 420 nm. Bars represent the average of 3 measurements, ± standard deviation. (0.71 MB TIF) [file pone.0014564.s002.tif]

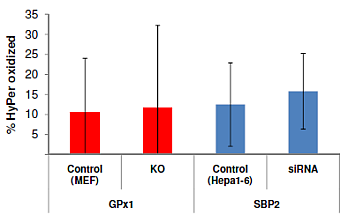

Supplement: Figure S3 — Redox state of cytosolic HyPer expressed in different cell types. Percentage of oxidized HyPer in MEF cells derived from GPx1 knockout mice (left) and siRNA SBP2 Hepa1-6 stable cell line (right). Cells were trypsinized, washed and diluted in PBS (pH 7.4). Fluorescence intensity ratio was measured for untreated cells (basal conditions), treated with 1 mM DTT for 30 min (fully reduced state) or 100 µM H2O2 (oxidized state) for 5 min. Fluorescence intensity ratio was obtained using emission at 530 nm and excitations at 500 nm and 420 nm. (0.23 MB TIF) [file pone.0014564.s003.tif]

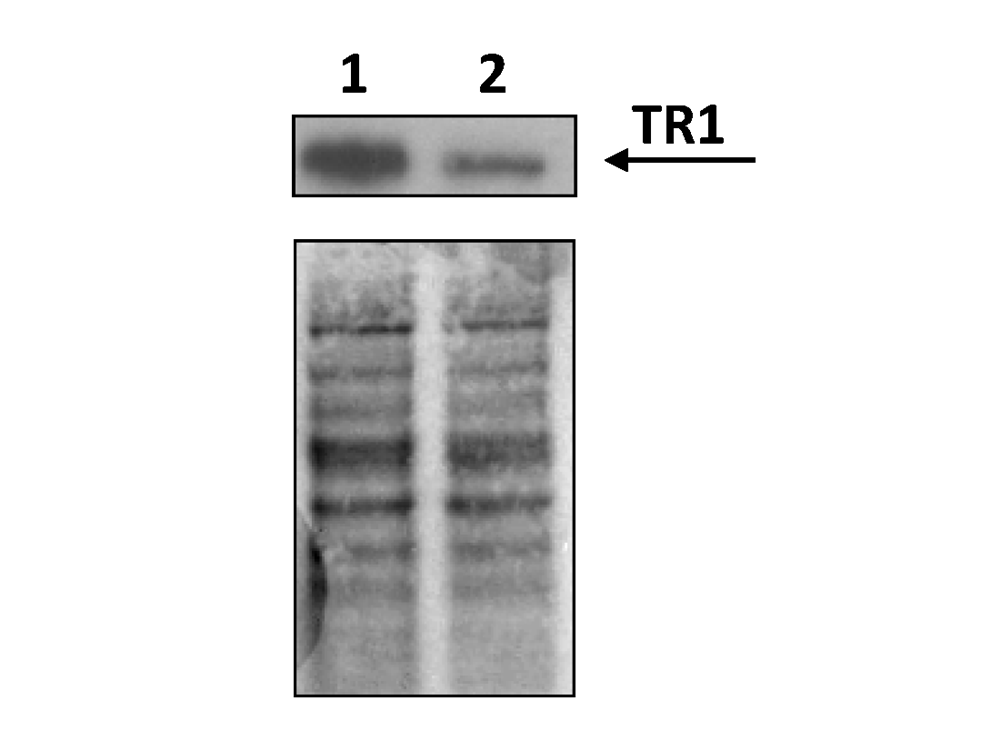

Supplement: Figure S4 — Expression of TR1 in TCMK1 cells. The upper panel shows immunoblot analysis of TR1 expression in TCMK1 cells (lane 1) and TCMK1 siTR1 cells (lane 2). The lower panel shows protein staining as a loading control. (0.22 MB TIF) [file pone.0014564.s004.tif]

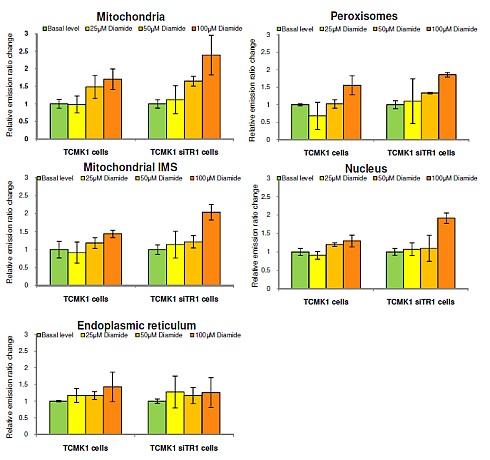

Supplement: Figure S5 — Redox state of HyPer targeted to different compartments in TR1 knockdown TCMK1 cells treated with diamide. TR1 knockdown and control TCMK cells stably expressing HyPer in the cytosol were treated for 30 min with indicated concentrations of diamide. Fluorescence intensity ratio was obtained using emission at 530 nm and excitations at 500 nm and 420 nm. Data represent mean of 3 measurements, ± standard deviation. (0.68 MB TIF) [file pone.0014564.s005.tif]

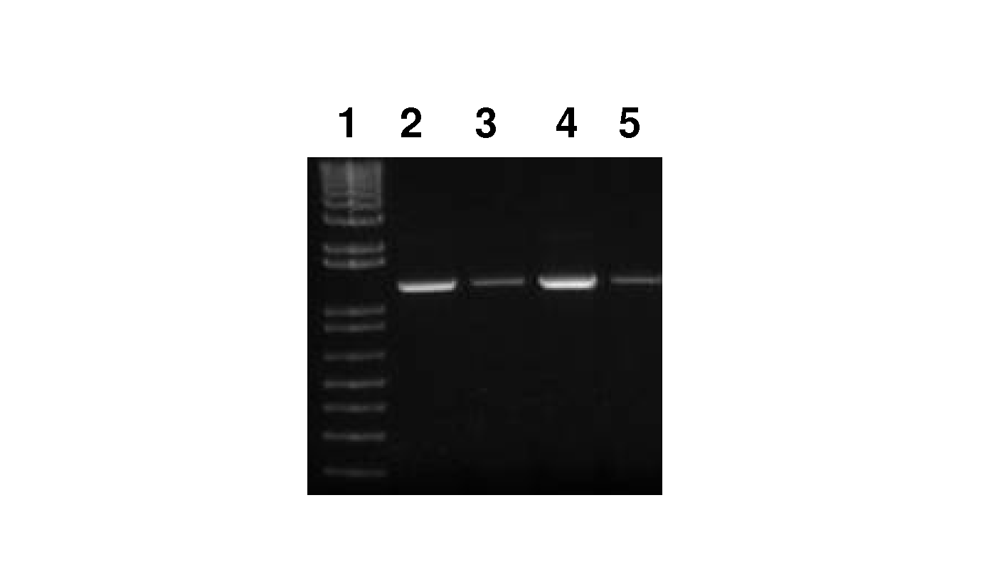

Supplement: Figure S6 — Ero1 mRNA levels in knockdown cells. mRNA was isolated from Ero1 siRNA-transfected and scrambled siRNA-transfected HEK 293 cells and subjected to semi-quantitative RT-PCR. Lanes represent RNA isolated from cells transfected with scrambled siRNA (lanes 2 and 4) and Ero1 siRNA (lanes 3 and 5). Molecular weight markers are shown in lane 1. (0.10 MB TIF) [file pone.0014564.s006.tif]

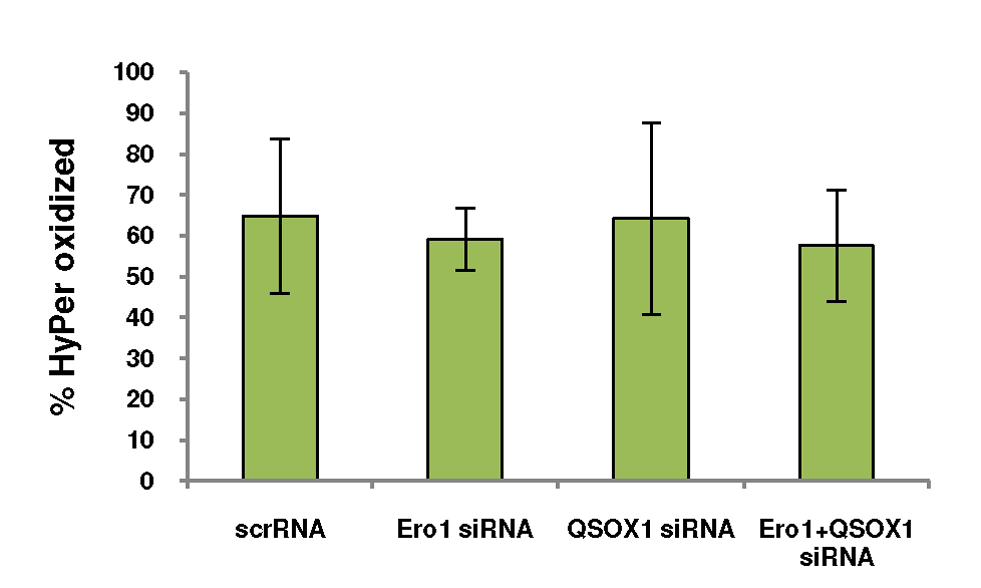

Supplement: Figure S7 — Knockdown of Ero1 or QSOX1 does not affect redox state of HyPer in HEK 293 cells. HEK 293 cells stably expressing HyPer in the ER were transfected with control (non-targeting), Ero1 or QSOX1 siRNAs. Fluorescence intensity was recorded 48 h after transfection. In addition, cells were treated with 1 mM DTT and 100 µM H2O2 to determine the range of oxidation states of HyPer in cells. Data represent mean of 3 independent measurements. (0.12 MB TIF) [file pone.0014564.s007.tif]

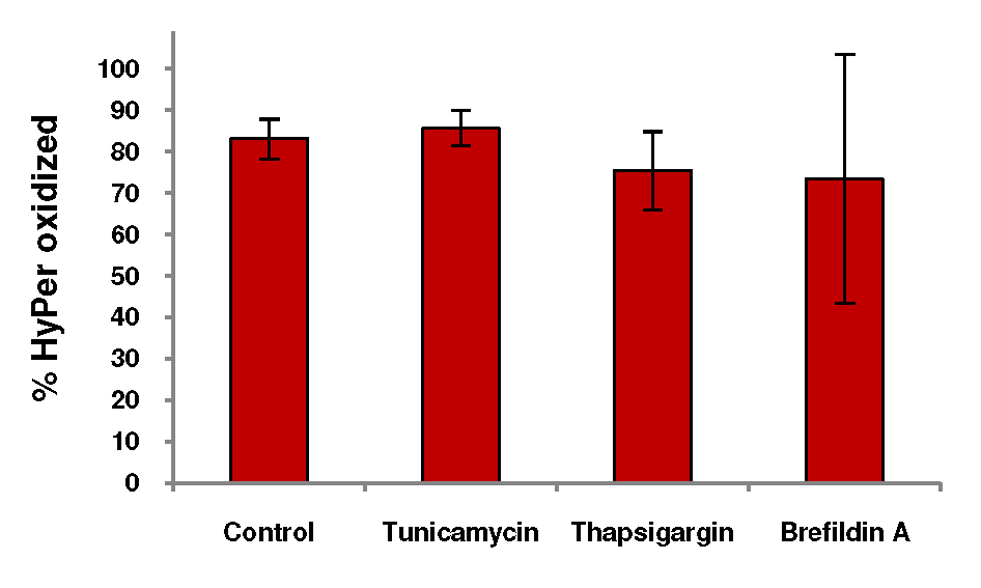

Supplement: Figure S8 — HyPer response to activators of ER stress. HEK293 cells stably expressing HyPer in the ER were treated with 2 µg/µl tunicamycin, 5 µg/µl brefeldin A or 2 µM thapsigargin for 6 h. In addition, cells were treated with 1 mM DTT and 100 µM H2O2 to determine the range of oxidation states of HyPer in cells. Data represent mean of 3 measurements, ± standard deviation. (0.12 MB TIF) [file pone.0014564.s008.tif]
