# Supplementary material for: Hydrogen Peroxide Probes Directed to Different Cellular Compartments
Source: PLoS One. 2011 Jan 21;6(1):e14564. doi: 10.1371/journal.pone.0014564 (PMC3024970; doi:10.1371/journal.pone.0014564)
Supplement: Table S1 — Primers used to create constructs expressing HyPer targeted to various cellular compartments. (0.01 MB DOCX) [file pone.0014564.s009.docx]

| **Construct** | **Primers** |
| --- | --- |
| Endoplasmic reticulum HyPer | **Pr1** 5’-AGATCCGCTAGCATGAGCATCCTACTGTCGCCGCCGTCGCTGCTGCTGCTTCTTGCAGCCCTTGTGGCTCCAGCCACCTCCGCCACCATGGAGATGGCAAGC -3’ |
|  | **Pr2** 5’-TTCCGTGGATCCTTACAGCTCGTCCTTAACCGCCTGTTTTAAAACTTTCAGCTCGTCCTT-3’ |
| Peroxisomal HyPer | **Pr3** 5’- TGAACCGTCAGATCCGCTAGC 3’ |
|  | **Pr4** 5’- TTCCGTGGATCCTTACAGCTTGCTAACCGCCTGTTTTAAAACTTT -3’ |
| Mitochondrial intermembrane space HyPer | **Pr5** 5’- AGATCCGCTAGCATGGCGTTTCAAAAGGCAGTG -3’ |
|  | **Pr6** 5’- GGATCTGGATCCATCGATGTCTGAAGGCAGCAG -3’ |
| Nuclear HyPer | **Pr7** 5’- CTTTTTTGGATCTACCTTTCTCTTCTTTTTTGGATCAACCGCCTGTTTTAAAAC -3’ |
|  | **Pr8** 5’- TTAAGTGGATCCTTATGCGGCTACCTTTCTCTTCTTTTTTGGATCTACCTTTCT -3’ |
