# Supplementary material for: Hydrogen Peroxide Probes Directed to Different Cellular Compartments
Source: PLoS One. 2011 Jan 21;6(1):e14564. doi: 10.1371/journal.pone.0014564 (PMC3024970; doi:10.1371/journal.pone.0014564)
Supplement: Table S2 — Redox state of HyPer in various cellular compartments of HEK 293 cells. (0.01 MB DOCX) [file pone.0014564.s010.docx]

| **Cellular compartment** | **% HyPer oxidized** |
| --- | --- |
| Cytosol | 12±23 |
| Mitochondria | 16±6 |
| Mitochondrial IMS | 14±19 |
| Endoplasmic Reticulum | 69±14 |
| Peroxisomes | 10±7 |
| Nucleus | 0.4±6 |
